# Polygenic risk of paclitaxel-induced peripheral neuropathy: a genome-wide association study

**DOI:** 10.1186/s12967-022-03754-4

**Published:** 2022-12-06

**Authors:** Kosar Hooshmand, David Goldstein, Hannah C. Timmins, Tiffany Li, Michelle Harrison, Michael L. Friedlander, Craig R. Lewis, Justin G. Lees, Gila Moalem-Taylor, Boris Guennewig, Susanna B. Park, John B. Kwok

**Affiliations:** 1 School of Medical Sciences, Faculty of Medicine and Health, The University of Sydney, Camperdown, NSW Australia; 2 Brain and Mind Centre, Faculty of Medicine and Health, The University of Sydney, Camperdown, NSW Australia; 3 Prince of Wales Clinical School, University of New South Wales, Sydney, NSW Australia; 4 Chris O’Brien Lifehouse, Camperdown, NSW Australia; 5 School of Biomedical Sciences, University of New South Wales, UNSW Sydney, Sydney, NSW Australia

**Keywords:** Paclitaxel, Chemotherapy-induced Peripheral Neuropathy, Axon, Genome-wide association study

## Abstract

**Background:**

Genetic risk factors for chemotherapy-induced peripheral neuropathy (CIPN), a major dose-limiting side-effect of paclitaxel, are not well understood.

**Methods:**

We performed a genome-wide association study (GWAS) in 183 paclitaxel-treated patients to identify genetic loci associated with CIPN assessed via comprehensive neuropathy phenotyping tools (patient-reported, clinical and neurological grading scales). Bioinformatic analyses including pathway enrichment and polygenic risk score analysis were used to identify mechanistic pathways of interest.

**Results:**

In total, 77% of the cohort were classified with CIPN (n = 139), with moderate/severe neuropathy in 36%. GWAS was undertaken separately for the three measures of CIPN. GWAS of patient-reported CIPN identified 4 chromosomal regions that exceeded genome-wide significance (rs9846958, chromosome 3; rs117158921, chromosome 18; rs4560447, chromosome 4; rs200091415, chromosome 10). rs4560447 is located within a protein-coding gene, *LIMCH1*, associated with actin and neural development and expressed in the dorsal root ganglia (DRG). There were additional risk loci that exceeded the statistical threshold for suggestive genome-wide association (*P* < 1 × 10^–5^) for all measures. A polygenic risk score calculated from the top 46 ranked SNPs was highly correlated with patient-reported CIPN (r^2^ = 0.53; *P* = 1.54 × 10^–35^). Overlap analysis was performed to identify 3338 genes which were in common between the patient-reported CIPN, neurological grading scale and clinical grading scale GWAS. The common gene set was subsequently analysed for enrichment of gene ontology (GO) and Reactome pathways, identifying a number of pathways, including the axon development pathway (GO:0061564; *P* = 1.78 × 10^–6^) and neuronal system (R-HSA-112316; adjusted *P* = 3.33 × 10^–7^).

**Conclusions:**

Our findings highlight the potential role of axon development and regeneration pathways in paclitaxel-induced CIPN.

**Supplementary Information:**

The online version contains supplementary material available at 10.1186/s12967-022-03754-4.

## Background

Paclitaxel is a highly active chemotherapeutic agent used widely in the treatment of solid tumours [1]. However, chemotherapy-induced peripheral neurotoxicity (CIPN) is a major dose-limiting neurological side-effect of paclitaxel treatment that can persist long-term [2]. CIPN produces sensory and functional abnormalities leading to difficulties with fine motor and balance tasks, increased falls risk, and reduced quality of life [3]. Further, CIPN is a common cause of dose reduction and premature discontinuation, potentially affecting survival outcomes [4]. There are currently no neuroprotective measures to prevent the development of CIPN and no effective treatment options [5]. Importantly, understanding the mechanisms underlying CIPN and identifying which patients are most at-risk are critical to preventing long-term sequelae of treatment with paclitaxel.

Mechanistically, paclitaxel targets microtubules, inhibiting the dynamic assembly and disassembly of β-tubulin, leading to their stabilisation, cell-cycle arrest, and cell death [1]. While this mechanism has been proposed to produce neurotoxicity via disruption to axonal transport [6], growing evidence suggests a range of additional mechanisms, including disruption of neuronal cell metabolism, mitochondrial dysfunction, oxidative stress and neuroinflammation as underlying the development of CIPN [7]. Better understanding of underlying mechanisms of CIPN will be critical to the development of successful preventative and treatment strategies.

There have been substantial efforts to identify genetic profiles associated with heightened CIPN risk, with a range of single nucleotide polymorphisms (SNPs) in genes associated with neural development and structure, drug metabolism and neural repair associated with paclitaxel-induced CIPN [8]. However, there has been limited replication between studies, and there remains a lack of validated genetic associations with paclitaxel-induced CIPN. A key limitation is the lack of consensus regarding appropriate CIPN outcome measures [9]. It has been well documented that patients report greater neuropathy severity than clinicians [10] and that patient-reported outcomes and clinician-reported outcomes provide complementary but different information about CIPN [11]. However, despite this, there has been limited studies incorporating multimodal CIPN outcome measures with comprehensive phenotyping and patient-reported outcome measures.

Ultimately, identification and validation of genetic pathways involved in CIPN will enable characterisation of patients at-risk of significant, persistent toxicity. CIPN risk likely incorporates multiple genes [7] and polygenic models will be required to explain variability in CIPN outcomes rather than reliance on single SNPs. However, such models need to be developed in appropriately phenotyped cohorts. In the present study, we utilised comprehensive CIPN assessment and phenotyping using multiple assessment tools combined with genome-wide association studies (GWAS) and pathway analysis to provide an improved understanding of the genetic variants contributing to paclitaxel-induced CIPN.

## Methods

We performed a GWAS on 183 paclitaxel-treated patients with comprehensive neuropathy phenotyping. Bioinformatic analyses, including pathway enrichment and polygenic risk score analysis, were used to identify mechanistic pathways of interest.

### Participants and neuropathy assessment

Germline DNA samples, clinical details and detailed neuropathy phenotyping were collected from paclitaxel-treated patients enrolled in observational CIPN cohort studies at Australian cancer centres. Patients assessed for neuropathy status following completion of paclitaxel-based treatment were eligible. Data relating to cancer diagnosis and treatment were recorded from medical records. Ethical approval was granted by the Sydney Local Health District and South-Eastern Sydney Local Health District Human Research Ethics Committees. All patients provided written informed consent to participate.

Patients underwent a clinical and functional CIPN assessment following the completion of paclitaxel chemotherapy. Multiple methods were used to quantify CIPN, including the clinical grading scale National Cancer Institute Common Terminology Criteria for Adverse Events (NCI-CTCAE) version 4.0 sensory neuropathy subscale, which graded CIPN severity as Grade-0 ‘no symptoms’, 1 ‘asymptomatic, not interfering with daily function’, 2 ‘moderate symptoms, limiting daily function’, 3 ‘severe symptoms, limiting daily function and self-care’, and 4 ‘disabling’. The neurological grading scale Total Neuropathy Score–clinical version (TNSc © Johns Hopkins University)[12] was utilized and incorporated six domains (sensory symptoms, motor symptoms, upper and lower limb pinprick and vibration sensibility, lower limb strength, deep tendon reflexes) graded from 0 to 4 (most severe presentation), for a maximum score of 24.

The patient-reported outcome measure European Organization for Research and Treatment of Cancer Quality of Life Questionnaire-Chemotherapy-Induced Peripheral Neuropathy (EORTC QLQ-CIPN20) [13] was utilized and included 20 symptom questions, each rated from “not at all” (1) to “very much” (4) before summation and linear transformation to a 0–100 scale, with higher numbers representing greater CIPN.

### Genotyping and quality control

DNA was extracted from blood and genotyped using the Illumina INFINIUM Microarray on GlobalScreeningAssay-24, with coverage of ~ 654,027 fixed markers. Quality control procedures and GWAS were implemented in the free, open‐source whole‐genome association analysis toolset PLINK version1.9 and R statistical software. Following sample quality control, individuals with a heterozygosity rate > 0.03% or sex discrepancy were excluded. SNPs with poor genotype clustering performance, excess missingness > 1%, minor allele frequency < 1%, and out of Hardy Weinberg equilibrium proportions < 1e^−6^ were removed, leaving 289,351 SNPs for subsequent analysis. The multidimensional scaling approach was used for the correction of population stratification using the 1000 Genomes Project data.

### Bioinformatic and statistical analyses

Clinical correlation analysis was undertaken in GraphPad Prism version 9.3.1 for Windows (GraphPad Software, California USA), using Mann–Whitney U tests or Spearman’s correlation coefficients. Normality was assessed using the Shapiro–Wilk test and results were presented as mean and standard deviation or median (interquartile range IQR) or for normally distributed and non-normally distributed data, respectively.

### Genome-wide association studies

Within PLINK, linear regression models were fit to predict the association between SNPs and CIPN using continuous variables related to phenotypes of interest**.** Correlation analyses indicated that age and body mass index (BMI) were significantly associated with all measures of CIPN and were included as co-variates in the subsequent GWASs. Quantile–quantile (Q-Q) plots of the marginal asymptotic *P* values were evaluated for the remaining population stratification. Sanity checks of individual variants were conducted in SPSS software package v.26.0 to confirm results. SNPs with nominal *P* values of ≤ 5 × 10^–8^ were considered to exceed genome-side significance, and *P* values of < 1 × 10^–5^ were considered suggestive for genome-wide association. LocusZoom was used to generate Manhattan plots and higher resolution plots of top associated SNPs [14].

### PRS analysis

The *P* values and effect sizes (Beta values) from the top-ranked SNPs identified from the EORTC-QLQ-CIPN20 GWAS were used to calculate the polygenic risk score (PRS) using the PRSice package version 2.3.2 [15]. PRS were computed for a range of *P* value thresholds from the GWAS top-ranked SNPs. A correlation (r^2^) *P* value was calculated between an individual’s PRS and their patient-reported CIPN for each defined P-value threshold in order to identify the optimal panel of SNPs based on the association between the PRS and phenotype. The PRSice algorithm also adjusted for linkage disequilibrium between the SNPs using the Clump function to identify index SNPs used to calculate the PRS.

### Candidate variant analysis

We included variants that were reported in previous GWAS studies to be significantly associated with paclitaxel-induced neuropathy for replication using our dataset. LDlinkR [16] computational software was interrogated to identify the optimal proxy variant (r^2^ < 0.1) with our dataset and to determine whether a SNP of interest lies in a potential regulatory genomic region. LDlinkR, which contains data from the 1000 Genomes Project, searches for proxy and putatively functional variants by exploring linkage disequilibrium (LD) structure in a native R environment.

### Pathway enrichment analysis

The PASCAL (Pathway scoring algorithm) [17] was used to provide insight into the biological processes in terms of gene-based *P* values by aggregating the association signal from GWAS analysis while correcting for LD structure using 1000 Genomes Project data [18]. Functional enrichment (Over-Representation Analysis (ORA)) of Geneontology (Biological Process) and Pathways (Reactome) in our list of genes commonly identified by all three measures of neuropathy was performed using WebGestalt (WEB-based Gene SeT AnaLysis Toolkit).

## Results

### Clinical characteristics

A total of 183 paclitaxel-treated patients were included in the GWAS analyses (Table 1). The median age was 59 years (range 27–85 years). The majority were breast cancer (n = 103, 56%) or ovarian cancer (n = 41, 23%) patients who completed their paclitaxel-based chemotherapy (median cumulative dose 960 (IQR 240) mg/m^2^) at a median of 6 months prior to assessment (range 0–59 months). 78 patients received concurrent carboplatin (n = 77) or cisplatin (n = 1).

**Table 1:** Clinical and demographic characteristics of 183 paclitaxel-treated patients

Clinical features	Median (IQR) or n (%)
Age, years	59 (18)
Female	179 (97.8%)
BMI, kg/m2	26.0 (8.2)
Time since treatment, months	6 (7)
Diabetes	15 (8.2%)
Cancer Type	
Breast	103 (56.2%)
Ovarian	42 (23%)
Endometrial	25 (13.7%)
Other (Primary peritoneal serous papillary carcinoma, gastrointestinal, Testis, Larynx, Oesophagus, Cervical)	13/183 (7.1%)
Cancer Stage	
0/1	19 (10.4%)
2	58 (31.7%)
3	64 (35%)
4	26 (14.2%)
Unknown	16 (8.7%)
Paclitaxel treatment	
Cumulative paclitaxel dose, mg/m2	960 (240)
Ceased paclitaxel due to neurotoxicity	45 (24.5%)
NCI-CTCAE grades	
Grade 0	44 (24%)
Grade 1	74 (40.4%)
Grade 2	55 (30.1%)
Grades 3/4	10 (5.5%)
Neurological grading scale (TNSc)	3 (4)
Patient reported CIPN (EORTC-QLQ-CIPN20)	9.3 (15.8)

In total, 77% of the cohort were classified with CIPN (n = 139), with moderate/severe CIPN in 36% (NCI-CTCAE grade 2 + ; n = 65; Additional file 1: Fig. S1). All three measures of CIPN were correlated with each other (NCI-CTCAE and TNSc r = 0.60; NCI and EORTC-QLQ-CIPN20 r = 0.77; TNSc and EORTC-QLQ-CIPN20 r = 0.62; all *P* < 0.0001). There was no significant difference in CIPN severity by platinum-treatment status (NCI-CTCAE *P* = 0.628; TNSc *P* = 0.799; or EORTC-QLQ-CIPN20 score *P* = 0.598) or time since treatment (≥ 6 vs < 6 months post completion of paclitaxel; NCI-CTCAE *P* = 0.538; TNSc *P* = 0.98; EORTC-QLQ-CIPN20 *P* = 0.726). All three measures of CIPN were significantly associated with age (NCI-CTCAE r = 0.400; TNSc r = 0.401; EORTC-QLQ-CIPN20 r = 0.395; all *P* < 0.0001) and BMI (NCI-CTCAE r = 0.216; TNSc r = 0.217; EORTC-QLQ-CIPN20 r = 0.283; all *P* < 0.001).

### GWAS identifies four chromosomal regions significantly associated with patient-reported CIPN

**Fig. 1:**
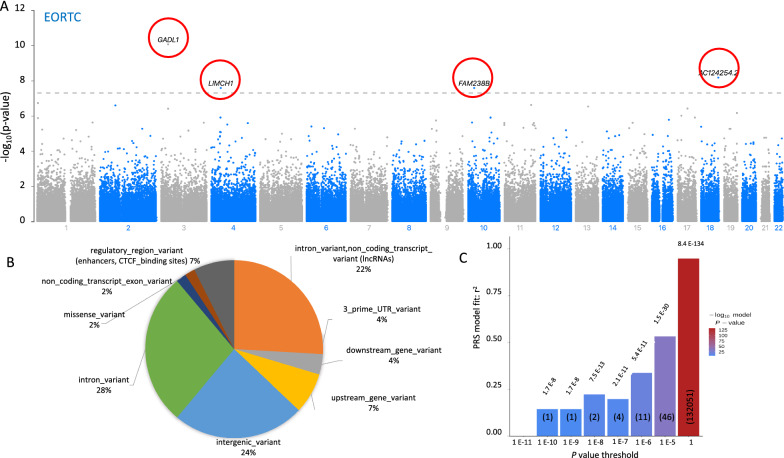
**A**) Manhattan plot showing the unadjusted *P-*value for the association of all SNPs with patient-reported CIPN (EORTC-QLQ-CIPN20). GWAS identified four loci that exceeded genome-wide significance of *P* < 5 × 10^–8^ (red circles). **B**) Distribution of top associated SNPs from EORTC-QLQ-CIPN20 GWAS (*P* < 10^–5^ cut-off) into functional categories including known genes, non-coding RNAs and intergenic variants within regulatory elements. **C**) Bar plot for PRS calculated for each *P*-value range and their correlation with the patient-reported scores. The corresponding number of SNPs that fall within each threshold is indicated in brackets

GWAS was undertaken separately for the three measures of CIPN. Notably, the GWAS of patient-reported CIPN (EORTC-QLQ-CIPN20) identified 4 chromosomal regions that exceeded genome-wide significance (Fig. 1A and Table 2). In addition, there were risk loci that exceeded the statistical threshold for suggestive genome-wide association (*P* < 1 × 10^–5^) for patient-reported CIPN as well as for both clinical CIPN (NCI-CTCAE) and neurologically graded CIPN (TNSc) (Table 2). Q-Q plots of the expected and observed *P* values from the GWAS of the three measures of CIPN were generated (Additional file 1: Fig. S2). The 50th percentile genomic control lambda values were ~ 1, indicating that there were no underlying population stratifications in the patient cohort. The functional consequence of the top associated SNPs (*P* < 10^–5^) for the patient-reported CIPN GWAS, annotated using Ensembl Variant Effect Predictor (VEP) platform [19], indicated that the majority of SNPs (69%) mapped to known genes, of which 14/37 (38%) were within non-coding RNAs (Fig. 1B, Additional file 2: Table S1). Of note, 18 of these SNPs were considered intergenic (10 kb distal to known genes). Four of the intergenic variants were located within known regulatory elements known as enhancers (rs7536740, rs111669817, rs117378411 and rs728169) Additional file 2: Table S1), while another two intergenic SNPs (rs75263049 and rs77573336) had unknown functions, but had Combined Annotation Dependent Depletion (CADD) scores > 10..

**Table 2:** Genetic loci identified by the four measures of CIPN exceeding suggestive and genome-wide significance

Chromosome	Base position	SNP	Beta	P-Value	CIPN Measure
3	31064705	rs9846958	48.83	8.81E−11*	Patient-reported CIPN (EORTC QLQ-CIPN20)
18	75143190	rs117158921	27.44	6.65E−09 *	
4	41447510	rs4560447	73.28	2.60E−08 *	
10	26947753	rs200091415	73.28	2.60E−08 *	
1	4314360	rs16841032	34.79	1.86E−07	
11	115,685,183	rs116038987	30.96	2.37E−07	
2	67726417	rs79369145	28.28	2.54E−07	
13	72495513	rs150199484	22.06	2.93E−07	
17	43935838	rs2301689	8.405	3.81E−07	
3	31028952	rs1395163	33.93	3.89E−07	
19	55,896,795	rs3810167	14.01	6.83E−07	
17	17246862	rs60566375	20.2	9.84E−07	
4	40232733	rs115791832	20.94	1.24E−06	
17	75540158	rs73997920	45.74	1.24E−06	
10	97171050	rs11818044	26.71	1.25E−06	
10	97174537	rs7081076	26.71	1.25E−06	
10	97189291	rs76700761	26.71	1.25E−06	
1	4338586	rs75263049	29.1	1.33E−06	
16	74334369	rs118029597	32.16	1.66E−06	
9	24971701	rs117703887	20.41	1.82E−06	
1	239257873	rs74973152	21.46	2.36E−06	
10	17449757	rs12248657	36.51	2.37E−06	
3	184475364	rs4686398	44.55	2.42E−06	
15	40126543	rs275760	31.67	2.45E−06	
15	40143910	rs275729	31.67	2.45E−06	
11	252649	rs6540	19.47	2.51E−06	
11	258397	rs474787	19.47	2.51E−06	
11	127101270	rs117097754	44.46	2.54E−06	
4	157936227	rs77573336	28.35	2.57E−06	
4	69706215	rs62300681	31.36	3.10E−06	
11	122641495	rs4936743	17.25	3.17E−06	
11	211482	rs2293168	16.22	3.25E−06	
11	211841	rs75256197	16.22	3.25E−06	
11	130309871	rs111669817	25.6	3.58E−06	
6	22118002	rs114044180	31.04	3.98E−06	
18	1375378	rs111376654	19.95	4.12E−06	
11	82568940	rs12294147	20.89	4.73E−06	
15	25520032	rs117465857	30.75	4.94E−06	
6	75965294	rs45596238	23.44	4.98E−06	
11	134945120	rs77234116	35.36	5.09E−06	
2	182965672	rs16822577	23.36	5.40E−06	
7	19,868,985	rs117016159	25.2	5.41E−06	
19	24217139	rs117117437	20.71	5.46E−06	
19	28225540	rs117635212	20.71	5.46E−06	
9	7036643	rs117691749	23.4	5.48E−06	
1	22346009	rs2501296	21.81	6.00E−06	
1	168472921	rs7536740	16.05	6.34E−06	
12	111254708	rs117378411	27.25	6.54E−06	
3	118337935	rs77999651	17.92	6.85E−06	
9	27336982	rs74621663	17.26	6.89E−06	
14	37217129	rs8021974	12.66	7.62E−06	
4	38725705	rs728169	26.98	8.14E−06	
4	41372088	rs116183417	18.49	8.37E−06	
10	101214126	rs77000635	26.89	8.76E−06	
6	24445829	rs9358767	1.777	2.56E−06	Neurological grading (TNSc)
13	22776823	rs61945320	3.59	2.89E−06	
17	7931282	rs7217076	2.778	5.74E−06	
1	87930889	rs6683030	1.077	9.93E−06	
13	103476981	rs80322894	−0.4543	4.61E−06	Clinical grading (NCI CTCAE)
7	48093679	rs10254800	−0.1962	4.65E−06	
19	56169604	rs3786648	−0.2651	6.88E−06	
4	2058475	rs382939	−0.3386	7.99E−06	
5	99023153	rs115135785	−0.3333	8.68E−06	
1	153317515	rs41308407	−0.7946	8.80E−06	

^*^exceeded genome-wide significance

### Fine mapping of patient-reported CIPN GWAS loci and eQTL co-localisation

A high-resolution view of the genomic landscape of the four SNPs from the GWAS of patient-reported CIPN (EORTC-QLQ-CIPN20) that achieved genome-wide significance and the closest known genes was undertaken via LocusZoom (Additional file 1: Fig. S3). Potential expression quantitative trait loci (eQTL) within each top associated chromosomal region were identified on the basis of available gene expression data for the tibial nerve tissue as in [20] and RNA-Seq data for the DRG [21] (Table 2).

As shown in Additional file 1: Fig. S3A, rs4560447 on chromosome 4 is located within a protein-coding gene, *LIMCH1*, which encodes the LIM and calponin homology domains-containing protein associated with actin stress fibres [22]. Interrogation of Genotype-Tissue Expression (GTEx) database [23] using the LDexpress Tool [24], confirmed that a proxy variant for rs4560447 (rs79278739 with D’ = 1.0 and r^2^ = 0.817) is a significant eQTL for *LIMCH1* in coronary artery tissue, but no eQTL were detected for the tibial nerve (Table 3). However, *LIMCH1* is expressed in the DRG with the FPKM (Fragments Per Kilobase of transcript per Million mapped reads) value of 16.9.

Another 2 top associated variants, rs117158921 on chromosome 18 and rs200091415 on chromosome 10, mapped to two long non-coding RNAs (LncRNA) *AC124254.2* and *FAM238B,* respectively (Additional file 1: Fig. S3B and C, Table 3). The function of these two LncRNAs are currently unknown. There were no proxy eQTLs for rs117158921, while for rs200091415 a proxy variant (rs79208020 with D’ = 0.749 and r^2^ = 0.119) was identified for the *decaprenyl diphosphate synthase subunit 1* (*PDSS1*) gene and another variant (rs72481178 with D’ = 0.592 and r^2^ = 0.344) for *FAM238B*. *PDSS1* is minimally expressed in DRG (FPKM of 1.56), while no expression data was available for *FAM238B*. No tibial nerve eQTLs were identified for rs9846958 on chromosome 3, and the closest gene is *glutamate decarboxylase like 1* (*GADL1)* (Additional file 1: Fig. S3D), which had minimal expression in the DRG (FPKM of 0.09).

**Table 3:** Summary of in silico functional analysis on LD (*r*2 ≥ 0.6) block for top associated SNPs

SNP	rs9846958	rs117158921	rs4560447	rs200091415
Closest gene	*GADL1*	*AC124254.2*	*LIMCH1*	*FAM238B*
SNPs in LD ^28^	2	0	18	27
eQTL	None	None	*LIMCH1*	*PDSS1*, *FAM238B*
Expression in dorsal root ganglia	Minimal	ND	Yes	Minimal for PDSS1

### Candidate SNP analysis

Based on prior GWAS studies examining paclitaxel-induced CIPN (Additional file 1: Table S2) [20, 25–28], we performed focused candidate gene analyses which only included variants with significant association (at suggestive genome-wide significance level of p < 10^–5^) between the polymorphism and CIPN (Additional file 3: Table S3). No variants were identified with *P* values that exceeded correction for multiple testing.

### Polygenic risk scores generated from patient-reported CIPN GWAS

We examined the effect of multiple variants by calculating a polygenic risk score (PRS) comprising of SNPs with *P* values from the GWAS falling within identified thresholds (ranging from *P* > 10^–10^; 10^–8^, 10^–7^, 10^–6^, 10^–5^ and 1; Fig. 1C). A PRS calculated from 11 top-ranked variants (Table 1) with *P* values < 10^–6^, after adjusting for variants in linkage disequilibrium, was significantly correlated (r^2^ = 0.34; *P* = 5.36 × 10^–20^) with patient-reported CIPN (EORTC-QLQ-CIPN20 scores). The inclusion of a further 35 SNPs in the calculation of the PRS improved the association (r^2^ = 0.53; *P* = 1.54 × 10^–35^) between risk score and the patient-reported measure of CIPN (Fig. 1C).

### Gene-based and pathway analyses relevant to all three measures of CIPN

**Fig. 2:**
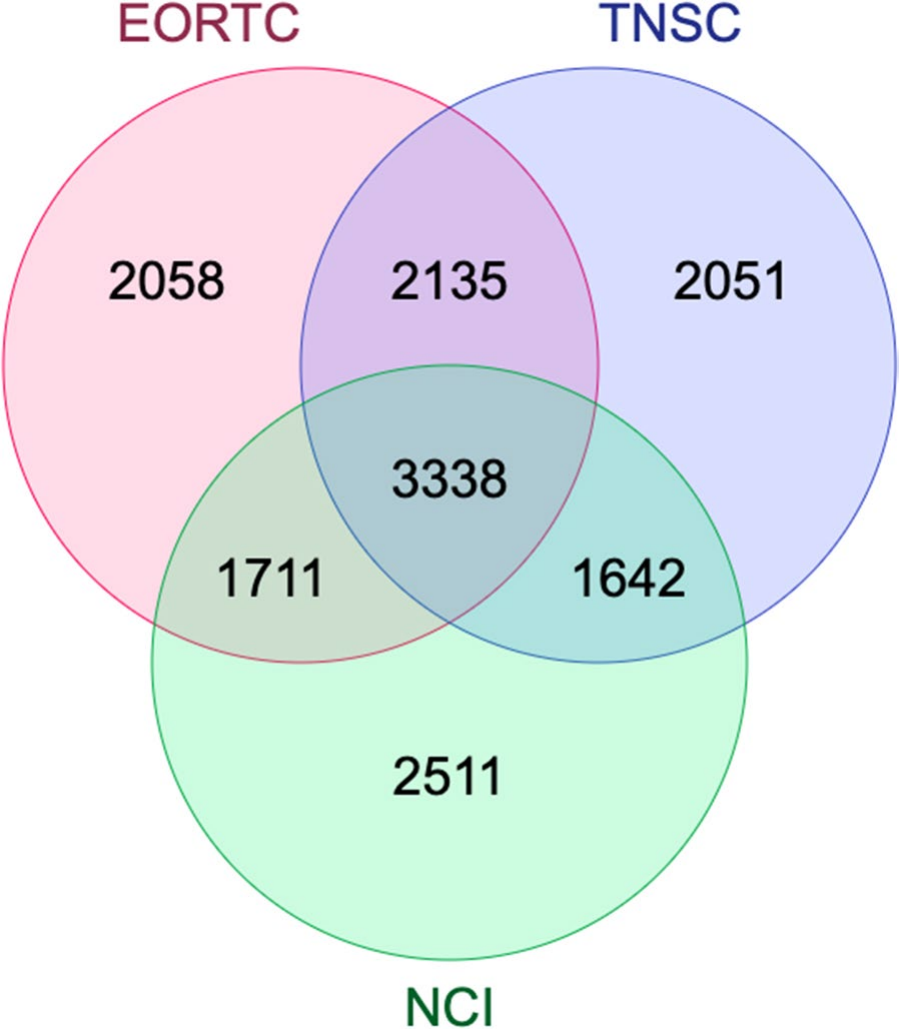
Overlap of gene lists derived from GWAS of the three measures of CIPN identifies 8827 genes common to at least 2 measures, and 3338 genes in common to all three measures

In order to identify common pathways relevant across different measures of CIPN, we calculated gene-based *P* values using PASCAL to yield a list of loci with gene-based *P* values of < 0.01 derived from the GWAS of each measure of CIPN. Overlap analysis was performed to identify n = 3338 genes which were in common between the patient-reported CIPN, neurological grading scale and clinical grading scale GWASs (Fig. 2). The common gene set was subsequently analysed for enrichment of gene ontology (GO) and Reactome pathways [29]. There were 10 GO terms that were significantly over-represented (Table 4, False discovery rate < 0.05), including the glutamate receptor signalling pathway (GO:0007215, enrichment ratio 2.8, adjusted *P* value = 3.85 × 10^–7^) and axon development (GO:0061564, enrichment ratio 1.65, adjusted *P* value = 1.78 × 10^–6^). Similarly, there were ten Reactome pathways that were significantly over-represented (Table 4), including Na^+^/Cl^−^ dependent neurotransmitter transporters (R-HSA-442660, enrichment ratio 4.4, adjusted *P* = 0.00033152) and Neuronal system (R-HSA-112316, enrichment ratio 1.9, adjusted *P* = 3.33 × 10^–7^).

**Table 4:** Over-Representation Analysis of Gene-ontology biological process terms and Reactome pathways associated with CIPN

Gene Set	Description	Enrichment Ratio	P-Value	FDR^a^
Gene-ontology Biological Process Terms				
GO:0007215	glutamate receptor signaling pathway	2.8127	4.21E−10	3.58E−07
GO:0034765	regulation of ion transmembrane transport	1.6919	5.54E−09	1.7765E−06
GO:0061564	axon development	1.645	8.01E−09	1.7765E−06
GO:0003013	circulatory system process	1.6516	8.36E−09	1.7765E−06
GO:0001655	urogenital system development	1.7983	1.18E−08	2.0012E−06
GO:0099177	regulation of trans-synaptic signaling	1.6614	5.31E−08	7.0381E−06
GO:0006898	receptor-mediated endocytosis	1.8105	5.80E−08	7.0381E−06
GO:0048880	sensory system development	1.6889	1.91E−07	0.000020307
GO:0030900	forebrain development	1.6521	3.53E−07	0.000033325
GO:0042391	regulation of membrane potential	1.593	9.14E−07	0.000077663
Reactome Pathways				
R-HSA-112316	Neuronal System	1.8569	1.92E−10	3.33E−07
R-HSA-382551	Transport of small molecules	1.5466	2.12E−09	1.8316E−06
R-HSA-5576891	Cardiac conduction	2.2029	1.02E−07	0.000058763
R-HSA-109582	Hemostasis	1.5141	1.76E−07	0.000064244
R-HSA-983712	Ion channel transport	2.004	1.96E−07	0.000064244
R-HSA-397014	Muscle contraction	1.9434	2.23E−07	0.000064244
R-HSA-112315	Transmission across Chemical Synapses	1.8549	6.46E−07	0.00015938
R-HSA-442660	Na ^+^ /Cl^-^ dependent neurotransmitter transporters	4.3595	1.53E−06	0.00033152
R-HSA-1474244	Extracellular matrix organization	1.697	1.84E−06	0.00035267
R-HSA-76002	Platelet activation, signaling and aggregation	1.7388	2.72E−06	0.00047018

^**a**^False Discovery Rate

## Discussion

Chemotherapy-induced peripheral neurotoxicity is a significant adverse event of paclitaxel treatment that can lead to early treatment discontinuation, persistent functional disability and reduced quality of life [2, 3]. In this study, we performed a GWAS on 183 patients treated with paclitaxel to identify genetic variants associated with CIPN, as measured using multiple neuropathy outcome measures. We identified multiple SNPs with genome-wide significance associated with patient-reported neuropathy. Pathways analysis was used to identify mechanistic pathways involved in CIPN and a polygenic risk score was determined. Importantly, our findings highlight the potential role of axon development and regeneration pathways in paclitaxel-induced CIPN.

Our study identified 4 chromosomal regions (rs9846958, nearest gene *GADL1* on chromosome 3; rs117158921, nearest gene *AC124254.2* on chromosome 18; rs4560447, nearest gene *LIMCH1* on chromosome 4; rs200091415, nearest gene *FAM238B* on chromosome 19) that passed genome-wide significance in the patient-reported neuropathy GWAS (*P* < 5 × 10^–8^; Table 2). Prior GWAS (E5103 (26), CALGB 40101 [25, 27, 28] and a meta-analysis of two GWAS studies (CALGB 40502 and CALGB 40101[20]) on patients treated with paclitaxel have identified a range of SNPs associated with neuropathy, but none exceeded genome-wide significance. In our study, the potential impact of top associated variants on the function of non-coding RNAs was highlighted by VEP annotation (Fig. 1B, Additional file 2: Table S1). This is consistent with results from a transcriptomic study that identified dysregulation of long non-coding RNAs and mRNAs mediating neuroinflammation and pain in the spinal cord of a rat model of paclitaxel-induced peripheral neuropathy [30].

While functional annotations have traditionally focused on known genes, thousands of disease-associated SNPs are located within intergenic regions, making it difficult to understand their association with disease phenotypes. Recent analyses found that non-coding disease associated SNPs were frequently located in or approximate to regulatory elements, such as the binding sites for CCCTC-binding factors (CTCF) and enhancer elements that act distally to promote gene expression [31]. In our annotation of the top 54 associated SNPs (Additional file 2: Table S1), 4 were located within these regulatory elements. CADD scores are based on various genomic features derived from surrounding nucleotide sequences, gene model annotations, evolutionary constraints, epigenetic marks and functional predictions [32]. We observed that 2 intergenic SNPs had CADD scores greater than 10, that is they were ranked in the top 10% of all known variants likely to be deleterious (Additional file 2: Table S1). We also note that one of the top associated SNP (rs9846958) on chromosome 3 would be considered to be an intergenic SNP and currently lacks any functional annotation, but we have indicated the closet gene to be *GADL1* (Fig. 1A).

In the present study, of the 4 SNPs with genome-wide significance, the associated gene *LIMCH1* was most prominently expressed in the DRG, a key region implicated in CIPN pathogenesis. *LIMCH1* has been identified as a key regulator of actin-cytoskeleton remodelling, involved in cell migration [22]. Due to its role in cell migration and adhesion, *LIMCH1* has been associated with worse prognosis in multiple forms of cancer [33]. While *LIMCH1* has not been directly associated with nerve function, actin-cytoskeletal frameworks are critical in neuronal development, and axonal growth and actin-binding LIM domain proteins are important in axonal regeneration [34]. Another actin-binding protein LIMK2, which acts to regulate cell proliferation and migration, has also been linked to paclitaxel-induced CIPN in a prior GWAS [27].

Further, there is substantial evidence highlighting the potential role of actin cytoskeleton and axonal guidance pathways in paclitaxel-induced CIPN [35]. Comparison of differences in signalling pathways and gene co-expression between paclitaxel-treated patients with and without CIPN provided molecular evidence of the involvement of cytoskeletal and axonal morphology pathways in neuropathy development [35]. This included a suite of genes previously associated with paclitaxel-induced neuropathy, including the *EPHA* gene family linked to receptors for axonal grown and neural development [27, 28] and *FDG4*, a F-actin binding protein [29]. Of note, although no candidate variants were independently replicated in our GWAS dataset, there was some support for *EPHA5* (genotyped SNP rs3605041, *P* = 0.0021 for TNSc-GWAS), which encodes an ephrin receptor important in neurite growth during development [7].

In further support of the importance of axonal and cytoskeletal development pathways in paclitaxel-induced CIPN, a key gene-ontology pathway of interest from our analysis across multiple outcome measures was the axon development pathway. This underscores the results of previous analyses, which have highlighted this pathway as central to paclitaxel-induced PN [7, 36]. Consistent with our findings (Table 4), differential gene expression and pathway impact analysis identified significantly perturbed cytoskeleton- and axon morphology-related signalling pathways in patients treated with paclitaxel [35]. These pathways have recently been highlighted in conjunction with their links to Ras homolog family of guanosine triphosphate hydrolase (RhoGTPase) signalling pathways relevant to axon extension and cell mobility [7]. RhoGTPases are important in sensory neuronal development and outgrowth as well as axonal regeneration [37] and are linked to paclitaxel-induced PN development, including via LIM domain proteins [37].

Although our study has identified several variants with genome-wide significance, we did not independently replicate the findings of prior studies in our dataset, given the number of candidate variants examined. However, the top replicated variant was rs9332998 from the CIPN20-GWAS, a proxy for rs4646487 within the *CYP4B1* gene. The gene is part of the *CYP* genes set that modulate paclitaxel pharmacokinetics and similar genes have been associated with paclitaxel-induced CIPN in prior analyses [9]. Replication studies have often failed to confirm genetic associations in CIPN, potentially related to a lack of standardisation in outcome measures, with different thresholds for CIPN case identification affecting findings [9].

We utilised multiple CIPN outcome measures, including patient reported symptoms, clinical grading scale and neurological assessment. While there remains no gold standard CIPN assessment tool, evidence suggests that multimodal CIPN assessment incorporating both patient report and clinician assessment may present the most comprehensive information about neuropathy status [11]. However, only a minority of prior genetic risk factor studies have utilised patient-reported outcomes [38]. Importantly, patients typically report greater severity of symptoms than reported by clinicians [10] and this has been demonstrated to affect the identification of genetic risk factors for paclitaxel-induced CIPN [38]. Conversely, there has been criticism of relying solely on patient-reported CIPN assessment for biomarker studies, as patient report may be more variable and lack a consistent benchmark of severity compared to clinical assessment [39]. Of note, in this study, SNPs with genome-wide significance were only identified in the GWAS using patient reported CIPN. This may be related to the sensitivity of patient-reported outcomes for neuropathy but may also reflect limitations in more objective outcomes which do not always match with patient report [11].

Another factor that complicates the search for genetic variants associated with paclitaxel-induced PN is the likely polygenic inheritance, with multiple variants contributing to the risk of PN [36, 40]. It is likely that a large number of SNPs each contribute a small, additive risk to the development of paclitaxel-induced PN [36, 40]. Importantly, the use of polygenic risk scores (PRS) which aggregate the effects of multiple genetic variants across the human genome into a single score, have recently been shown to have predictive value for multiple common diseases such as breast cancer and diabetes [41]. Further, the integration of genetic information with non-genetic risk factors has been demonstrated to enhance the sensitivity and specificity of PRS as a clinical tool [42]. In our dataset, a PRS calculated from 46 SNPs was highly correlated with patient-reported CIPN (Fig. 1C). Our PRS differs from scores calculated for idiopathic neurodegenerative diseases such as Alzheimer’s disease [43] which typically require > 100,000 SNPs and have poorer predictive values with r^2^ < 0.1. This may reflect the fact that pharmacogenomic variants typically have stronger genetic effects compared with common disease-associated variants [44]. We also note that our PRS is calculated from patients of European descent, and validation of our PRS by other investigators should involve controlling for population stratification. This is especially important as the rate of severe CIPN may vary by ethnicity [9, 25]. Nonetheless, such an approach is likely to be beneficial for the prediction of CIPN and should form the basis for future genetic analyses of CIPN.

### Strengths and limitations

This study has identified several variants with genome-wide significance linked to paclitaxel-induced peripheral neuropathy. A strength of the study was the inclusion of multiple neuropathy assessment tools, including validated patient reported outcomes. However, a limitation of our GWAS is the sample size, which may affect statistical power. Our findings should be replicated in larger datasets, preferably with diverse populations. In addition, our sample included multiple treatment protocols and cancer types, heterogeneity which may affect the generalizability of results to specific cohorts. Accordingly, our loci and PRS require validation and replication in independent datasets, preferably with compatible CIPN outcome measures. It should be noted that lack of standardization in CIPN outcome measures across studies and in particular in large-scale clinical trials of neurotoxic agents has limited the ability for data from different studies to be meaningfully combined. Hopefully efforts to standardize outcome measures for CIPN will assist towards this aim.

## Conclusions

In conclusion, we have identified novel genetic loci associated with patient-reported paclitaxel- induced peripheral neuropathy and these findings provide further evidence for the involvement of axon development pathways in paclitaxel-induced CIPN. Our study highlights the importance of appropriate and patient-relevant CIPN outcome measures in defining the CIPN phenotype. In total, this study highlights the polygenic nature of CIPN risk, as definition of polygenic patterns of inheritance will be critical to ultimately enable genetic risk factors to become useful tools to predict patient risk in the clinic and improve patient quality of life following paclitaxel treatment.

## Supplementary Information

**Additional file 1:** **Table S2.** Prior Genome-wide association studies examining predictors of CIPN in paclitaxel-treated patients. **Figure S1.** Distribution of CIPN severity in the patient cohort assessed using NCI-CTCAE clinical grading scale. **Figure S2.** (A) Q-Q and (B) Manhattan plots for GWAS of the three measures of CIPN including clinical grading scale (NCI), neurological grading scale (TNSc) and patient report (EORTC). **Figure S3:** Genetic loci corresponding to top associated SNPs identified by GWAS of patient reported EORTC-QLQ-CIPN20 CIPN visualized using LocusZoom.

**Additional file 2:** **Table S1. **Annotation of SNPs using Ensembl Variant Predictor platform.

**Additional file 3:** **Table S3. **Candidate SNPs derived from previously reported associations.

## Data Availability

The datasets used and/or analysed during the current study are available from the corresponding author on reasonable request.

## Abbreviations

BMI: Body mass index
CADD: Combined Annotation Dependent Depletion
CIPN: Chemotherapy induced peripheral neuropathy
DRG: Dorsal root ganglia
EORTC QLQ-CIPN20: European Organization for Research and Treatment of Cancer Quality of Life Questionnaire-Chemotherapy-Induced Peripheral Neuropathy
eQTL: Expression quantitative trait loci
GTEx: Genotype-Tissue Expression
GWAS: Genome wide association study
NCI-CTCAE: National Cancer Institute Common Terminology Criteria for Adverse Events
PRS: Polygenic risk score
RhoGTPase: Guanosine triphosphate hydrolase
SNP: Single nucleotide polymorphism
TNSc: Total Neuropathy Score–clinical version
Q-Q plots: Quantile–quantile plots
VEP: Variant Effect Predictor
