# Supplementary material for: Polygenic risk of paclitaxel-induced peripheral neuropathy: a genome-wide association study
Source: J Transl Med. 2022 Dec 6;20:564. doi: 10.1186/s12967-022-03754-4 (PMC9724416; doi:10.1186/s12967-022-03754-4)
Supplement: Supplementary file 1 — Additional file 1: Table S2. Prior Genome-wide association studies examining predictors of CIPN in paclitaxel-treated patients. Figure S1. Distribution of CIPN severity in the patient cohort assessed using NCI-CTCAE clinical grading scale. Figure S2. (A) Q-Q and (B) Manhattan plots for GWAS of the three measures of CIPN including clinical grading scale (NCI), neurological grading scale (TNSc) and patient report (EORTC). Figure S3: Genetic loci corresponding to top associated SNPs identified by GWAS of patient reported EORTC-QLQ-CIPN20 CIPN visualized using LocusZoom. [file 12967_2022_3754_MOESM1_ESM.docx]

**Additional File 1**

**Figure S1.** Distribution of CIPN severity in the patient cohort (n=183), assessed using NCI-CTCAE clinical grading scale.

**Figure S2.** (A) Q-Q and (B) Manhattan plots for GWAS of the three measures of CIPN. There were no loci which exceeded genome-wide significance value of *P* < 5 x 10^-8^ (indicated by dashed line) for clinical grading scale (NCI-CTCAE) or neurological grading scale (TNSc) reported CIPN but four loci for the patient-reported CIPN (EORTC-QLQ-CIPN20) GWAS.

** Figure S3:** Genetic loci corresponding to top associated SNPs (green circles) identified by GWAS of patient reported EORTC-QLQ-CIPN20 CIPN visualized using LocusZoom.

**Table S2.** Prior Genome-wide association studies examining predictors of CIPN in paclitaxel-treated patients

| **Study** | **Type** | **Cohort** | **CIPN measure** | **Key identified SNPs** | **Genome wide significance** | **Outcomes** |
| --- | --- | --- | --- | --- | --- | --- |
| Baldwin et al 2012 | GWAS | 855 paclitaxel treated (CALGB 40101) | NCI CTCAE | *FZD3, EPHA5, FGD4 (rs7001034, rs7349683, rs10771973)* | No; 7 SNPs P < 10^-5^ | 1. Cumulative dose to Grade ≥2 PN 2. Max NCI grade |
| Schneider et al 2015 | GWAS | 3431 paclitaxel treated (ECOG-5103); 2906 paclitaxel or docetaxel treated (ECOG-1199) | NCI CTCAE | rs3125923, FCAMR (rs1856746) | No; 5 SNPs P < 10^-5^ | Case (Grade 2-4 PN): Control (others) |
| Leandro-Garcia et al 2013 | GWAS; Meta-analysis GWAS | 144 paclitaxel/carboplatin treated | NCI CTCAE | Downstream of *EPHA4 (rs17348202); EPHA6 rs301927, EPHA5 (rs1159057), LIMK2 (rs4141404*  *XKR4 rs4737264* | No; 25 SNPs marginal P < 10^-5^ | Cumulative dose to ≥Grade 2 CIPN |
| Komatsu et al 2015 | GWAS | 183 paclitaxel treated | NCI CTCAE | PTPMT1 (protein tyrosine phosphatase, mitochondrial 1) BCR (breakpoint cluster region), AIPL1 | No; 4 SNPs marginal P < 10^-5^ | Case (≥ Grade 2): Control (Grade 0) |
| Chua et al 2020 | GWAS meta analysis | 469 CALGB 40502 and 855 CALGB 40101 Paclitaxel/ nab-paclitaxel/ixabepilone treated | NCI CTCAE | rs74497159/*S1PR1*, rs10771973/*FGD4*, rs11076190/*CX3CL1*,  rs9623812/*SCUBE1*, rs2060717/*CALU* | No; 18 SNPs with P < 10^-5^ | Cumulative dose to ≥Grade 2 CIPN |
